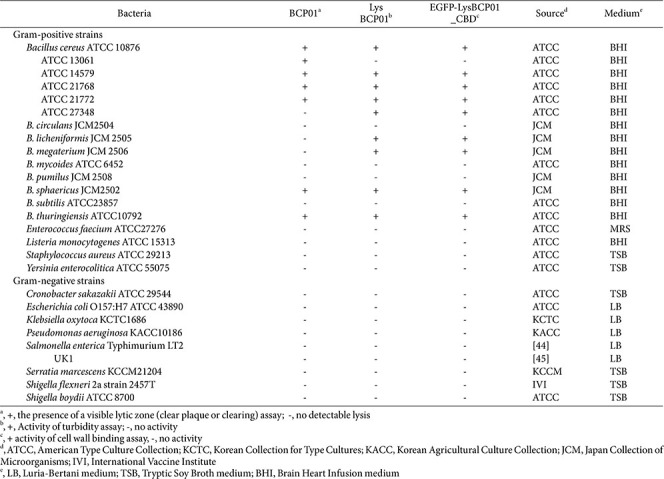# Bacteriophage BCP01 and Its Endolysin as Tools for Biocontrol and Rapid Detection of *Bacillus cereus*

**DOI:** 10.4014/jmb.2604.04006

**Published:** 2026-04-24

**Authors:** You-Tae Kim, Ji-Eun See, Ju-Hoon Lee

**Affiliations:** 1 Department of Agricultural Biotechnology, Seoul National University, Seoul 08826, Republic of Korea; 2 Department of Food and Animal Biotechnology, Seoul National University, Seoul 08826, Republic of Korea; 3 Center for Food and Bioconvergence, Seoul National University, Seoul 08826, Republic of Korea; 4 Department of Food Science and Biotechnology, Kyung Hee University, Yongin 17104, Republic of Korea

**Keywords:** *Bacillus cereus*, Bacteriophage, Endolysin, Biocontrol agent, Pathogen detection

## Abstract

*Bacillus cereus* is an important food-borne pathogen for which effective biocontrol and rapid detection strategies are needed. In this study, a novel *B. cereus*-infecting bacteriophage, BCP01, was isolated from sewage and characterized together with its phage-derived endolysin and cell wall-binding domain (CBD). BCP01 exhibited myoviral morphology, strong lytic activity against *B. cereus* ATCC 14579, and stability over a broad range of pH and temperature conditions. Genome analysis showed that BCP01 possessed a 157,958-bp double-stranded DNA genome containing 225 predicted open reading frames and no toxin- or virulence-associated genes. A putative endolysin gene, LysBCP01, encoding a modular protein with an N-terminal amidase domain and a C-terminal SH3_5 domain, was identified and heterologously expressed. Recombinant LysBCP01 showed broader lytic activity than the parental phage against several *Bacillus* strains and retained activity over a broad range of pH and temperature conditions. In food matrix assays, BCP01 reduced *B. cereus* counts in sterile cabbage, whereas LysBCP01 reduced viable cells in both cabbage and milk. To evaluate detection applicability, the CBD of LysBCP01 was fused to enhanced green fluorescence protein (EGFP). The resulting EGFP-LysBCP01_CBD showed a host-binding range identical to the lytic spectrum of LysBCP01 and enabled fluorescence-based detection of *B. cereus* in milk within 5 min. These findings suggest that bacteriophage BCP01 provides multifunctional phage-derived components applicable to both biocontrol and rapid detection of food-borne *B. cereus*.

## Introduction

*Bacillus cereus* is an important food-borne pathogen that poses a persistent threat to food safety and public health [1]. As a spore-forming bacterium, *B. cereus* can survive under harsh environmental conditions and persist in food-processing environments, thereby increasing the risk of contamination throughout the food chain [2]. In addition, it produces a variety of toxins responsible for emetic and diarrheal syndromes, making it a major concern in diverse food products [3]. Conventional control strategies, including antibiotics and chemical disinfectants, often show limited efficacy in complex environments such as food matrices and industrial settings [4, 5]. Moreover, the emergence and dissemination of multidrug-resistant bacteria, together with concerns regarding the safety, environmental impact, and possible cross-resistance associated with disinfectant overuse, have highlighted the need for alternative antimicrobial approaches that are both effective and sustainable [6, 7].

Bacteriophages (phages), viruses that specifically infect bacteria, have gained renewed attention as promising biocontrol agents against bacterial pathogens [8]. Phages offer several advantages, including high host specificity, self-amplification in the presence of susceptible hosts, and minimal disruption of beneficial microbiota [9, 10]. These properties make them attractive candidates for applications in food safety, agriculture, and environmental hygiene [11]. In addition to their direct antibacterial activity, phages and phage-derived components have also been investigated as highly specific tools for rapid bacterial detection [12, 13]. By exploiting the highly selective interaction between phages and their host bacteria, phage-based platforms can enable sensitive and rapid identification of target pathogens [14]. Therefore, bacteriophages are increasingly regarded as versatile biological tools for both the control and detection of food-borne bacteria [13].

Among phage-derived antimicrobial proteins, endolysins have emerged as particularly promising alternatives [15]. Endolysins are phage-encoded peptidoglycan hydrolases that are synthesized during the late stage of the lytic cycle and degrade the bacterial cell wall to release progeny phage particles [16]. In Gram-positive bacteria, whose peptidoglycan layer is directly exposed to the extracellular environment, exogenously applied endolysins can readily access their substrate and exert potent bactericidal activity [17]. Most endolysins targeting Gram-positive bacteria possess a modular architecture consisting of an enzymatically active domain (EAD) and a cell wall binding domain (CBD), which together determine catalytic efficiency and host specificity [18]. Compared with whole phages, recombinant endolysins offer several practical advantages, including rapid lytic activity, a lower probability of resistance development due to their targeting of essential cell wall structures, and ease of production and engineering as antimicrobial proteins [15]. Furthermore, endolysins often exhibit broad lytic activity within closely related bacterial species and may act synergistically with other antimicrobial agents, including antibiotics and bacteriocins [19, 20].

Despite these advantages, the practical application of phages and endolysins is strongly influenced by their biological and biochemical characteristics, such as host range, thermal stability, pH tolerance, salt sensitivity, and activity in complex food-associated environments [7, 21]. Because these properties vary substantially depending on the phage-host system, the isolation of novel bacteriophages and detailed characterization of their lytic proteins are essential for developing effective phage-based biocontrol and detection strategies [13, 22]. In the present study, we isolated and characterized a novel *B. cereus*-infecting bacteriophage, BCP01. A putative endolysin gene was identified from the phage genome, cloned, and heterologously expressed, and the recombinant protein was subsequently evaluated for its lytic activity, host range, and biochemical stability. In addition, the applicability of phage BCP01 for rapid detection of *B. cereus* was assessed. Collectively, this study provides fundamental insights into the potential use of bacteriophage BCP01 and its endolysin as effective tools for the control and detection of food-borne *B. cereus*.

## Materials and Methods

### Bacterial Strains, Culture Conditions, and Reagents

The bacterial strains used in this study are listed in Table 1. *B. cereus* ATCC 14579 was used as the host strain for the isolation, propagation, and characterization of bacteriophage BCP01. All of the medium components used in this study were purchased from Difco (USA) and used in accordance with the instructions. All *Bacillus* strains and *Listeria monocytogenes* were grown with shaking in Brain Heart Infusion (BHI) broth at 37°C. *Staphylococcus*, *Yersinia*, *Cronobacter*, *Serratia*, and *Shigella* strains were grown with shaking in Tryptic Soy Broth (TSB) at 37°C. *Escherichia coli*, *Klebsiella*, *Pseudomonas*, and *Salmonella* strains were grown with shaking in Luria-Bertani (LB) broth at 37°C. *Enterococcus faecium* was grown in de Man-Rogosa-Sharpe (MRS) broth at 37°C under anaerobic conditions. Solid media were prepared by supplementing the corresponding broth media with agar. For bacteriophage propagation and plaque assays, soft agar overlays were prepared using the appropriate medium supplemented with 0.4% agar. For host range analysis, all bacterial strains were cultivated under the corresponding growth conditions described above. Unless otherwise specified, reagents used in this study were purchased from Sigma-Aldrich (USA).

### Isolation, Propagation, and Purification of Bacteriophage BCP01

Sewage samples were collected from the Seongnam Water Restoration Center (Republic of Korea) and used for the isolation of *B. cereus*-infecting bacteriophages. Bacteriophage isolation and propagation were performed as previously described with minor modifications [23]. For phage propagation, *B. cereus* ATCC 14579 was grown in BHI broth at 37°C with shaking until the optical density at 600 nm (OD_600_) reached approximately 0.5. A single plaque was picked and added to the host culture, followed by incubation at 37°C for 12 h with vigorous shaking. The lysate was then centrifuged at 6,000 × *g* for 20 min to remove bacterial cells, and the supernatant was passed through a 0.45-μm pore-size filter (Pall Co., USA).

To concentrate phage particles, the filtered lysate was mixed with polyethylene glycol (PEG) 6000 to a final concentration of 10% in the presence of 1 M NaCl and incubated overnight at 4°C. The precipitated phage particles were collected by centrifugation at 10,000 × *g* for 20 min and resuspended in sodium chloride-magnesium sulfate (SM) buffer containing 100 mM NaCl, 10 mM MgSO_4_·7H_2_O, and 50 mM Tris-HCl (pH 7.5). For further purification, the concentrated phage suspension was subjected to cesium chloride (CsCl) density gradient ultracentrifugation using step gradients of 1.3, 1.45, 1.5, and 1.7 g/mL at 78,500 × *g* and 4°C for 2 h (Optima XE; Beckman Coulter, USA). The visible phage band was collected using a sterile syringe and dialyzed against SM buffer using a Spectra/Por 4 dialysis membrane (Spectrum Laboratories Inc., USA). Purified phage particles were stored at 4°C until further use.

### Morphological and Biological Characterization of Bacteriophage BCP01

The morphology of bacteriophage BCP01 was examined by transmission electron microscopy (TEM) as previously described [23]. Purified phage particles were negatively stained with uranyl acetate and observed using a transmission electron microscope. Phage morphology was classified according to the guidelines of the International Committee on Taxonomy of Viruses. The host range of BCP01 was determined by a spot assay using the bacterial strains listed in Table 1, as previously described with minor modifications [23]. Briefly, exponentially grown bacterial cells were mixed with 0.4% soft agar and overlaid onto BHI agar plates, and serially diluted phage suspensions were spotted onto the bacterial lawn. After incubation at 37°C, lytic activity was evaluated based on plaque formation. The antibacterial activity of BCP01 against *B. cereus* ATCC 14579 was evaluated by a bacterial challenge assay. Exponentially growing host cells were treated with BCP01 at a multiplicity of infection (MOI) of 10, whereas SM buffer was added to the untreated control. Bacterial growth was monitored by measuring optical density at 600 nm, and viable cell counts were determined by plate counting. The stability of BCP01 under various temperature and pH conditions was assessed as previously described with minor modifications [23], and residual infectivity was determined by plaque assay using *B. cereus* ATCC 14579.

### Genomic DNA Extraction, Whole-Genome Sequencing, and Bioinformatic Analysis of BCP01

Genomic DNA of bacteriophage BCP01 was extracted as previously described [23] and subjected to whole-genome sequencing using the Illumina MiSeq platform (USA). Qualified sequence reads were assembled using CLC Genomics Workbench v10.0.1 (Qiagen, Germany). Open reading frames (ORFs) were predicted using Glimmer3, FgenesV (Softberry, Inc., USA), and GeneMarkS [24], and the predicted ORFs were further verified by ribosome-binding site analysis using RBSfinder [25]. Functional annotation of the predicted ORFs was performed using BLASTP and InterProScan [26, 27], and potential virulence-associated genes were screened using Virulence Searcher [28]. The complete genome sequence was visualized and analyzed using Artemis [29]. Based on the annotated genome sequence, putative lysis-related genes were selected for further *in silico* analysis.

### Identification and in silico Analysis of the Putative Endolysin and CBD Domains

A putative endolysin gene was identified from the annotated genome of bacteriophage BCP01 based on sequence similarity to known phage endolysins [30]. Conserved domains were analyzed using BLASTP and InterProScan [26, 27], and the enzymatically active domain (EAD) and cell wall-binding domain (CBD) were identified based on the predicted domain architecture. The full-length endolysin gene and the CBD region were then selected for subsequent cloning and recombinant expression.

### Cloning, Expression, and Purification of Recombinant LysBCP01 and EGFP-LysBCP01_CBD

The genes encoding LysBCP01 and its CBD domain were amplified by PCR using the genomic DNA of bacteriophage BCP01 as a template. For amplification of the full-length LysBCP01 gene, the primer set LysBCP01-F (5'-AAACATATGATGGGAACATATAACGTA-3') and LysBCP01-R (5'-AAACTCGAGTTACTGGAAAGTTCC-3') was used (underlined sequences signify restriction enzyme recognition sites). Restriction enzyme recognition sites were incorporated into the primers for directional cloning. The PCR product containing the full-length LysBCP01 gene was cloned into the pET15b expression vector (Novagen, USA) using the *NdeI* and *XhoI* restriction sites to generate an N-terminal His-tagged fusion protein. For the production of EGFP-LysBCP01_CBD, the PCR-amplified CBD region with the primer set CBD-F (5'-AAAAAAGGATCCT GGTTCACACCA-3') and CBD-R (5'-AAAAAACTCGAGTTAGATCCAAACGTAAC-3'), and then inserted into the pET15b vector using the *Bam*HI and *XhoI* restriction sites. The enhanced green fluorescent protein (EGFP) gene was subsequently inserted into the same vector using the *NdeI* and *Bam*HI restriction sites. The resulting constructs were transformed into *E. coli* BL21(DE3) for recombinant protein expression.

For protein expression, the transformants were cultured until the OD_600_ reached approximately 0.7 and then induced with 0.5 mM isopropyl-β-D-thiogalactopyranoside (IPTG). After incubation for 20 h at 18°C with shaking, the cells were harvested by centrifugation and resuspended in lysis buffer (50 mM Tris-Cl, 200 mM NaCl, pH 8.0). Cell disruption was performed by sonication under cold conditions, and the lysates were centrifuged to remove cell debris. The supernatants were then filtered through a 0.22-μm syringe filter and applied to HisPur Ni-nitrilotriacetic acid (NTA) chromatography cartridges (Thermo Scientific, USA). The recombinant proteins were eluted with buffer containing 300 mM imidazole and used for subsequent analyses.

### Lytic Activity and Host Range Analysis of Recombinant LysBCP01

The lytic activity of recombinant LysBCP01 was evaluated by a turbidity reduction assay using *B. cereus* ATCC 14579 as the indicator strain, as previously described with minor modifications [23]. Briefly, bacterial cells were harvested at the exponential growth phase, washed, and resuspended in an appropriate reaction buffer. Purified LysBCP01 was added at various concentrations, and the decrease in optical density at 600 nm was monitored over time. Lytic activity was expressed as the reduction in turbidity compared with that of the untreated control. In the dose-response assay, LysBCP01 was tested at final concentrations of 50, 100, and 500 μg/mL. The lytic spectrum of LysBCP01 was determined against the bacterial strains listed in Table 1 by the same turbidity reduction assay [23]. Relative lytic activity was evaluated based on the percentage decrease in OD_600_ after 8 min of treatment and was categorized as no lysis (−), limited lysis (+), moderate lysis (++), or rapid lysis (+++).

### Stability of BCP01, LysBCP01, and EGFP-LysBCP01_CBD under Various Conditions

The stability of bacteriophage BCP01, recombinant LysBCP01, and EGFP-LysBCP01_CBD under various temperature and pH conditions was evaluated as previously described with minor modifications [23]. BCP01 was tested over temperature and pH ranges of -20 to 50°C and pH 3 to 11, respectively, whereas LysBCP01 and EGFP-LysBCP01_CBD was tested over ranges of -20 to 60°C and pH 2 to 10. For pH stability, the pH of the reaction buffer was adjusted using HCl or NaOH prior to treatment. BCP01 was exposed to each condition for 12 h, whereas LysBCP01 and EGFP-LysBCP01_CBD were treated for 1 h. Residual infectivity of BCP01 was determined by plaque assay using *B. cereus* ATCC 14579 as the indicator strain. The lytic activity of LysBCP01 was assessed by turbidity reduction assay, and the binding activity of EGFP-LysBCP01_CBD was evaluated by a *B. cereus*-specific cell wall binding assay.

### Evaluation of Antibacterial Activity in Food Matrices

The antibacterial activities of bacteriophage BCP01 and recombinant LysBCP01 in food matrices were evaluated as previously described with minor modifications [23]. *B. cereus* ATCC 14579 was used as the indicator strain, and sterile cabbage samples (5 g) and sterilized commercial milk were selected as model food systems. The food samples were artificially contaminated with *B. cereus* ATCC 14579 to a final concentration of 10^5^ CFU/mL.

For evaluation of the antibacterial activity of BCP01 in food matrices, contaminated food samples were treated with purified phage suspension at the indicated MOIs, and viable bacterial counts were determined at the indicated time points by serial dilution and plating on BHI agar plates. The reduction in viable cell numbers compared with those of the untreated control was used to assess the antibacterial activity of BCP01 in food environments.

For evaluation of the antibacterial activity of LysBCP01, contaminated cabbage samples were incubated at room temperature for 1 h, followed by treatment with purified LysBCP01 at a final concentration of 1.1 mg/mL. The samples were further incubated at room temperature for up to 1 h and collected at 15-min intervals. Each sample was homogenized with sterile 0.1% peptone water for 30 s using a stomacher, and the homogenates were filtered, centrifuged, serially diluted in PBS, and plated on BHI agar for viable cell counting. For milk samples, LysBCP01 was added to a final concentration of 1.7 mg/mL, and the same procedure was used except for the homogenization step. Plates were incubated at 37°C overnight before enumeration.

### Rapid Detection of *B. cereus* Using EGFP-LysBCP01_CBD

The rapid detection capability of EGFP-LysBCP01_CBD was assessed by fluorescence-based cell wall-binding assays as previously described with minor modifications [23]. Briefly, bacterial cells grown to the exponential phase were collected, washed, and incubated with purified EGFP-LysBCP01_CBD. After removal of unbound fusion protein by washing, the cells were examined under a fluorescence microscope together with corresponding bright-field images. The host binding range of the fusion protein was evaluated using the bacterial strains listed in Table 1.

To evaluate its applicability in a food matrix, sterilized commercial milk contaminated with *B. cereus* ATCC 14579 at 10^5^ CFU/mL was treated with EGFP-LysBCP01_CBD. After 5 min of incubation, the sample was washed twice and observed under fluorescence microscopy. The detection of fluorescently labeled bacterial cells was used to assess the potential of EGFP-LysBCP01_CBD as a rapid detection probe for *B. cereus* in food samples.

### Statistical Analysis

All experiments were performed independently at least in triplicate, and the results are presented as the mean ± standard deviation. Statistical analyses were carried out using SPSS software. Differences among groups were analyzed by one-way analysis of variance (ANOVA) followed by Duncan’s multiple range test. A *p* value of < 0.05 was considered statistically significant.

## Results

### Biological Characterization of Bacteriophage BCP01

**Fig. 1:**
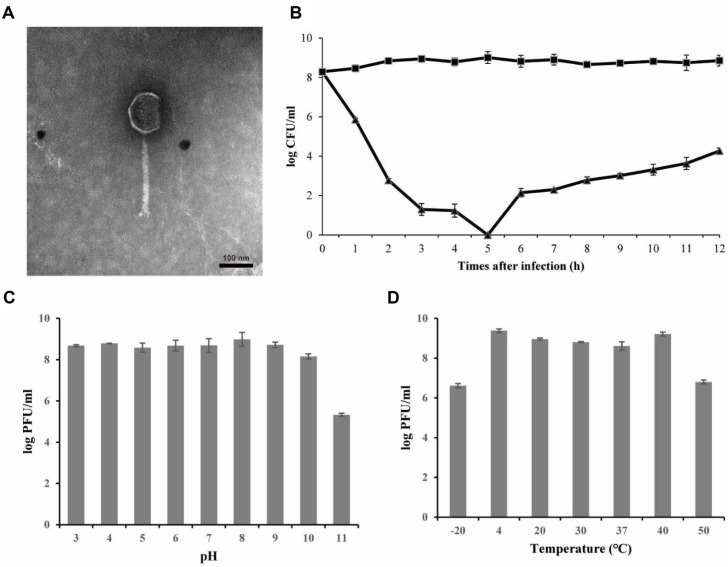
Morphological and biological characterization of bacteriophage BCP01. (**A**) Transmission electron micrograph of bacteriophage BCP01. Scale bar, 100 nm. (**B**) Bacterial challenge assay of BCP01 against *B. cereus* ATCC 14579 at a multiplicity of infection (MOI) of 10. Triangles ( ▲ ), BCP01-infected samples; squares ( ■ ), non-BCP01-infected samples. (**C**) pH stability of BCP01 determined using *B. cereus* ATCC 14579 as the indicator strain. (**D**) Temperature stability of BCP01 determined using *B. cereus* ATCC 14579 as the indicator strain.

Bacteriophage BCP01, isolated using *B. cereus* ATCC 14579 as the host strain, was examined by TEM, which showed that BCP01 possessed an icosahedral head and a contractile tail, consistent with myoviral morphology (Fig. 1A). The head diameter was approximately 114.4 ± 1.4 nm, and the tail length was approximately 225.0 ± 3.2 nm. The host range of BCP01 was evaluated against the bacterial strains listed in Table 1. Among the tested strains, BCP01 showed lytic activity against seven *Bacillus* strains, including five *B. cereus* strains, *B. thuringiensis*, and *B. sphaericus*, whereas no lytic activity was observed against the other tested *Bacillus* strains, non-*Bacillus* Gram-positive bacteria, or Gram-negative bacteria (Table 1). In particular, BCP01 lysed *B. cereus* ATCC 10876, ATCC 13061, ATCC 14579, ATCC 21768, and ATCC 21772, but not *B. cereus* ATCC 27348.

The lytic activity of BCP01 was assessed by an *in vitro* bacterial challenge assay using *B. cereus* ATCC 14579. Following phage treatment at an MOI of 10, the viable cell count of the host strain decreased compared with that of the untreated control. Approximately a 9-log-unit reduction was observed at 5 h after phage addition, and an approximately 4.6-log-unit reduction was maintained at 12 h in the phage-treated culture (Fig. 1B). The reduction in viable cell count was first clearly detected after 3 h of incubation and was maintained throughout the experimental period. The stability of BCP01 was evaluated under different pH and temperature conditions. BCP01 retained infectivity over a pH range of 3 to 11 and a temperature range of -20 to 50°C (Fig. 1C and 1D). Under pH 3-10, the phage titer remained at approximately 8.15 to 8.98 log PFU/mL, and a titer of 5.33 log PFU/mL was still detected at pH 11. Under the tested temperature conditions, the phage titer remained at approximately 8.61 to 9.38 log PFU/mL between 4 and 40°C, and 6.81 log PFU/mL was retained even after treatment at 50°C. No detectable infectivity was observed under more extreme pH and temperature conditions.

### Genome Analysis of BCP01 and Identification of LysBCP01

**Fig. 2:**
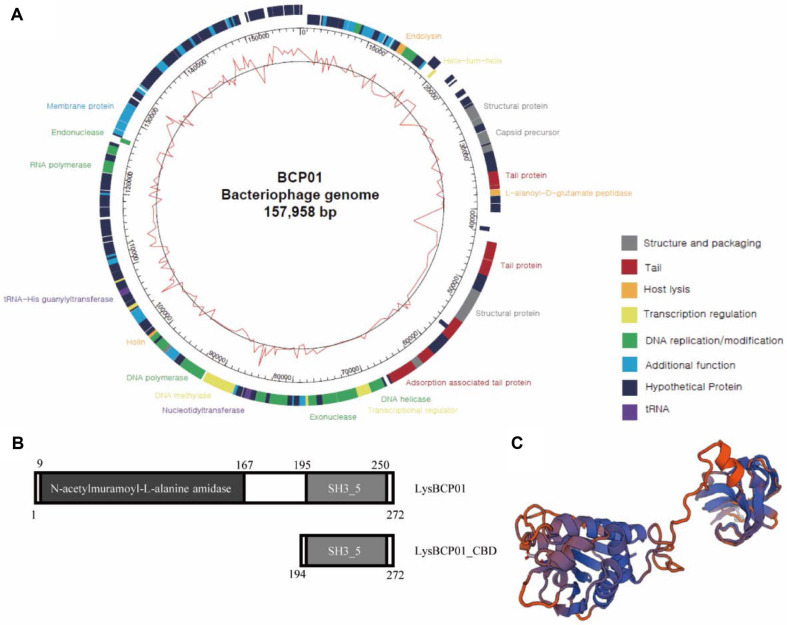
Genome analysis of bacteriophage BCP01 and identification of LysBCP01. (**A**) Genome map of bacteriophage BCP01. The inner circle indicates GC content, and the outer circle shows predicted open reading frames (ORFs) on each strand. Functional categories are indicated by different colors. (**B**) Schematic diagrams of LysBCP01 and LysBCP01_CBD. LysBCP01 contains an N-terminal Amidase_3 domain and a C-terminal SH3_5 domain, and LysBCP01_CBD includes the SH3_5 domain. (**C**) Predicted tertiary structure of LysBCP01.

The complete genome sequence of BCP01 was obtained by Illumina MiSeq sequencing and assembled into a single contig. The genome of BCP01 consisted of 157,958 bp of double-stranded DNA with a GC content of 40.34%, and 225 ORFs were predicted in the genome (Fig. 2A). Among these, 75 ORFs (33.33%) were assigned putative functions, whereas the remaining ORFs were annotated as hypothetical proteins (GenBank accession number MG602477). The functional ORFs were categorized into seven groups, including host lysis, DNA replication/modification, structure and packaging, tail, transcription regulation, tRNA, and additional functions. No toxin- or virulence factor-related gene was identified in the BCP01 genome (Fig. 2A).

Among the annotated ORFs, a putative endolysin-encoding gene was identified as ORF BCP01_029 and designated LysBCP01. The LysBCP01 gene was 816 bp in length and encoded a protein of 272 amino acids (Fig. 2B). Conserved domain analysis showed that LysBCP01 contained an N-terminal N-acetylmuramoyl-L-alanine amidase domain (Amidase_3) and a C-terminal bacterial SH3 domain (SH3_5). More specifically, the Amidase_3 domain was located at amino acid residues 9-167, whereas the SH3_5 domain was located at residues 195-250, with an internal linker region between residues 168 and 194 (Fig. 2B). The predicted tertiary structure of LysBCP01 showed two spatially separated domains connected by an extended linker region. The N-terminal region formed a larger globular domain with mixed α-helical and β-sheet elements, whereas the C-terminal region formed a smaller compact domain, consistent with the modular organization of LysBCP01 (Fig. 2C). Amino acid sequence alignment showed that LysBCP01 shared 96.7% amino acid sequence identity with a homologous protein from *Bacillus* phage TsarBomba (YP_009206875).

### Expression and Characterization of Recombinant LysBCP01

**Fig. 3:**
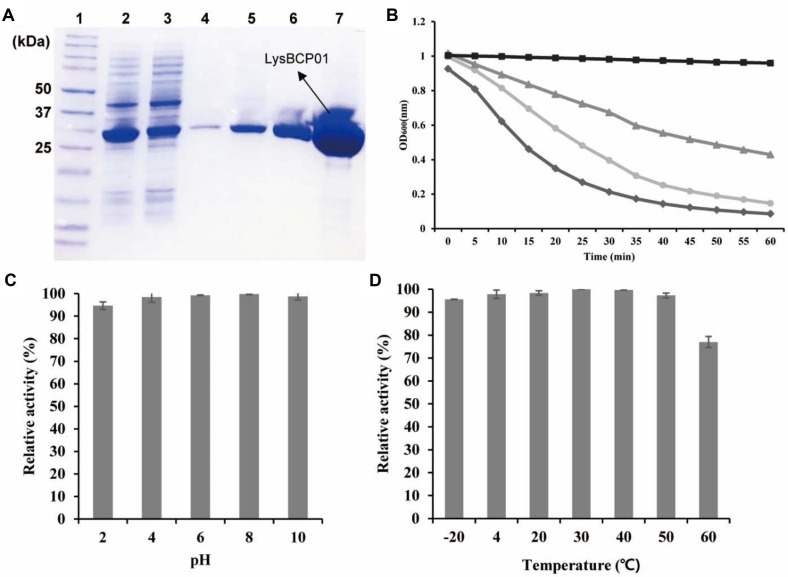
Expression and characterization of recombinant LysBCP01. (**A**) SDS-PAGE analysis of purified LysBCP01. Lane 1, size marker (Precision Plus Protein™ Dual Color Standards, Bio-Rad); lanes 2–3, Ni-NTA flow-through; lanes 4–5, washing fraction (25 mM imidazole); lanes 6–7, elution fraction (300 mM imidazole). (**B**) Turbidity reduction assay of LysBCP01 against *B. cereus* ATCC 14579. Different concentrations of LysBCP01 were added to bacterial suspensions, and the decrease in turbidity was monitored over time. Squares ( ■ ), non-endolysin-treated suspension; triangles ( ▲ ), 50 μg/mL LysBCP01-treated suspension; circles ( ● ), 100 μg/ml LysBCP01-treated suspension; diamonds ( ◆ ), 500 μg/mL LysBCP01-treated suspension. Stability of LysBCP01 under different pH (**C**) and temperature (**D**) conditions, determined using *B. cereus* ATCC 14579 as the indicator strain. Error bars indicate standard deviations from triplicate experiments.

The gene encoding LysBCP01 (BCP01_029) was cloned and overexpressed in *E. coli*, and the recombinant protein was purified by Ni-NTA affinity chromatography. SDS-PAGE analysis showed a single protein band at approximately 30 kDa, which was consistent with the calculated molecular mass of 29.92 kDa (Fig. 3A). The lytic activity of LysBCP01 was evaluated by turbidity reduction assay using *B. cereus* ATCC 14579. Treatment with LysBCP01 caused a concentration-dependent decrease in OD_600_, and the reduction in turbidity became more rapid as the protein concentration increased. Among the tested concentrations, 500 μg/ml showed the fastest decrease in OD_600_, followed by 100 μg/mL and 50 μg/mL (Fig. 3B).

The lytic spectrum of LysBCP01 was further examined against the bacterial strains listed in Table 1 by measuring the percentage decrease in OD_600_ within 8 min. LysBCP01 showed lytic activity against all BCP01-susceptible strains except *B. cereus* ATCC 13061. In addition, LysBCP01 lysed several strains that were not susceptible to BCP01, including *B. cereus* ATCC 27348, *B. licheniformis* JCM 2505, and *B. megaterium* JCM 2506 (Table 1). The relative lytic activity of LysBCP01 ranged from limited to rapid lysis depending on the test strain. The stability of LysBCP01 was examined under various pH and temperature conditions. LysBCP01 retained lytic activity over a pH range of 2 to 10 and a temperature range of -20 to 60°C (Fig. 3C and 3D). No detectable lytic activity was observed outside these ranges.

To further compare the contribution of individual domains to the lytic spectrum, two chimeric endolysins, BCP01EAD-PBC1CBD and PBC1EAD-BCP01CBD, were also constructed and purified (Fig. S1A and S1B). Both chimeric proteins showed lytic activity against selected *Bacillus* strains, but their lytic spectra were narrower than those of the parental endolysins LysBCP01 and LysPBC1 (Table S1).

### Antibacterial Activity of BCP01 and LysBCP01 in Food Matrices

**Fig. 4:**
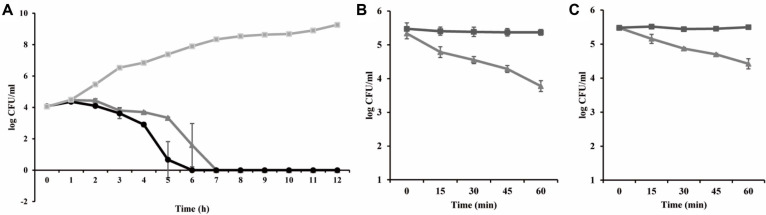
Antibacterial activities of BCP01 and LysBCP01 in food matrices. (**A**) Antibacterial activity of bacteriophage BCP01 in sterile cabbage artificially contaminated with *B. cereus* ATCC 14579. BCP01 was applied at MOIs of 10² and 10³, and viable bacterial cells were enumerated over time. Antibacterial activity of LysBCP01 in sterile cabbage (**B**) and sterilized commercial milk (**C**) artificially contaminated with *B. cereus* ATCC 14579. LysBCP01 was applied at final concentrations of 1.1 mg/mL in cabbage and 1.7 mg/ml in milk, and viable bacterial cells were determined at the indicated time points. Error bars indicate standard deviations from triplicate experiments.

The antibacterial activities of BCP01 and LysBCP01 were evaluated in representative food matrices artificially contaminated with *B. cereus* ATCC 14579. Sterile cabbage was used as a representative solid food matrix, whereas sterilized milk was additionally included to evaluate the activity of LysBCP01 in a liquid food system with different physicochemical properties. The antibacterial activity of BCP01 was evaluated in sterile cabbage, whereas that of LysBCP01 was examined in both sterile cabbage and sterilized commercial milk (Fig. 4). In sterile cabbage contaminated with *B. cereus*, treatment with BCP01 reduced the viable cell count by approximately 4 log CFU/mL. This reduction was observed within 6 h at an MOI of 10^3^ and within 7 h at an MOI of 10^2^, and the reduced cell counts were maintained up to 12 h after treatment (Fig. 4A). The antibacterial activity of the full-length lytic protein LysBCP01 was then examined in both sterile cabbage and sterilized commercial milk inoculated with *B. cereus* ATCC 14579 at 10^5^ CFU/mL. In sterile cabbage, treatment with LysBCP01 at 1.1 mg/mL reduced the viable cell count by 0.74, 0.98, 1.27, and 1.56 log CFU/mL after 15, 30, 45, and 60 min, respectively (Fig. 4B). In sterilized commercial milk, treatment with LysBCP01 at 1.7 mg/mL reduced the viable cell count by 0.40, 0.63, 0.77, and 1.05 log CFU/ml after 15, 30, 45, and 60 min, respectively (Fig. 4C).

### Expression and Detection Performance of EGFP-LysBCP01_CBD

**Fig. 5:**
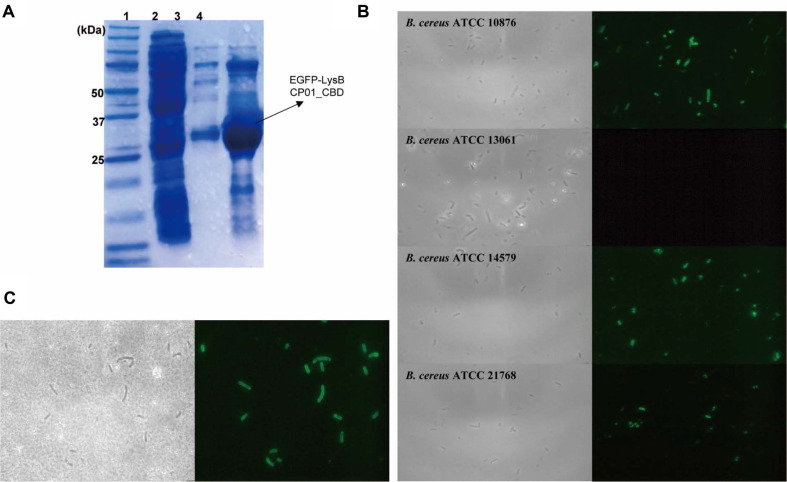
Expression and detection performance of EGFP-LysBCP01_CBD. (**A**) SDS-PAGE analysis of purified EGFP-LysBCP01_CBD. Lane 1, size marker (Precision Plus Protein™ Dual Color Standards, Bio-Rad); lane 2, Ni-NTA flow-through; lane 3, washing fraction (25 mM imidazole); lane 4, elution fraction (300 mM imidazole). (**B**) Binding activity of EGFP-LysBCP01_CBD observed by bright-field (left lane) and fluorescence microscopy (right lane). (**C**) Rapid detection of *B. cereus* ATCC 14579 in sterilized milk using EGFP-LysBCP01_CBD, observed by bright-field (left) and fluorescence microscopy (right).

To determine whether the CBD of LysBCP01 could function independently as a non-lytic host recognition module, the CBD region corresponding to amino acid residues 194-272 was fused to EGFP and expressed in *E. coli*. The resulting fusion protein, designated EGFP-LysBCP01_CBD, was purified by Ni-NTA affinity chromatography. SDS-PAGE analysis showed a single protein band at approximately 33.11 kDa, which was consistent with the expected size of the fusion protein (Fig. 5A). The host-binding activity of EGFP-LysBCP01_CBD was evaluated by fluorescence microscopy using the bacterial strains listed in Table 1. After treatment with the fusion protein, susceptible *Bacillus* strains showed clear fluorescent signals on the cell surface, whereas no fluorescence was observed in the non-target strains (Fig. 5B and Table 1). The binding range of EGFP-LysBCP01_CBD was identical to the lytic spectrum of LysBCP01, and fluorescent labeling was observed in *B. cereus* ATCC 10876, ATCC 14579, ATCC 21768, ATCC 21772, ATCC 27348, *B. licheniformis* JCM 2505, *B. megaterium* JCM 2506, *B. sphaericus* JCM2502, and *B. thuringiensis* ATCC 10792 (Table 1).

The stability of the binding activity of EGFP-LysBCP01_CBD was further examined under various pH and temperature conditions. The fusion protein retained binding activity over a pH range of 2 to 10 and a temperature range of -20 to 60°C (Supplementary Fig. S2A and S2B). Binding activity was not detected after exposure to more extreme pH or temperature conditions. The rapid detection performance of EGFP-LysBCP01_CBD was evaluated in sterilized milk artificially contaminated with *B. cereus* ATCC 14579 at 10^5^ CFU/mL. After incubation with the fusion protein for 5 min, the milk sample was washed twice and examined by fluorescence microscopy. Fluorescently labeled bacterial cells were detected in the contaminated milk sample after the washing steps (Fig. 5C).

## Discussion

In the present study, a novel *B. cereus*-infecting bacteriophage, BCP01, was isolated and characterized, and its potential applicability for both biocontrol and rapid detection was evaluated. BCP01 exhibited typical myoviral morphology, a narrow host range mainly restricted to *Bacillus* species, strong lytic activity against *B. cereus*, and stability over broad pH and temperature ranges. Genome analysis further identified a putative endolysin gene, LysBCP01, encoding a modular protein composed of an N-terminal amidase domain and a C-terminal SH3_5 cell wall-binding domain, which is a common structural feature of endolysins targeting Gram-positive bacteria [17, 31]. Recombinant LysBCP01 showed broader lytic activity than the parental phage against several *Bacillus* strains and retained antibacterial activity in food matrices. In addition, the isolated CBD of LysBCP01, when fused to EGFP, functioned as a host recognition module and supported its applicability for rapid detection of *B. cereus* in milk. BCP01 was evaluated as an intact bacteriophage for host infection and phage-based biocontrol, LysBCP01 as a full-length lytic protein for exogenous antibacterial application, and EGFP-LysBCP01_CBD as a non-lytic host recognition probe for fluorescence-based detection. Collectively, these findings support the potential applicability of phage-derived components from a single *B. cereus* phage for both antimicrobial control and bacterial detection in food-related environments.

BCP01 shares several general characteristics with previously described *B. cereus* group phages, including a myoviral morphology and host specificity largely confined to closely related *Bacillus* strains [32]. However, host range among *Bacillus* phages has been reported to vary substantially, from highly strain-specific phages such as PBC1, which infects only a very limited subset of *B. cereus* strains [30], to broader-host-range phages such as vB_BceM_Bc431v3 and Bcp1, which infect multiple members of the *B. cereus* *sensu lato* group [33, 34]. In this context, BCP01 appears to occupy an intermediate position, showing specificity mainly toward *Bacillus* species while remaining inactive against non-Bacillus Gram-positive and Gram-negative bacteria. Such selectivity may be advantageous for food biocontrol, because narrow host specificity can reduce disturbance to non-target bacteria present in complex microbial communities [9, 10]. In addition, whereas many reported *B. cereus* phages have been characterized primarily at the level of morphology, genome organization, and host range, some phages such as BCP1-1 and BCP8-2 have been further evaluated in food-related systems [35]. In that regard, the retained antibacterial activity of BCP01 in sterile cabbage extends its relevance beyond routine phage characterization and supports its consideration as an application-oriented phage candidate for selective control of *B. cereus* in food-associated environments.

In contrast to the relatively restricted host range of the intact phage, LysBCP01 exhibited lytic activity against a slightly broader set of *Bacillus* strains, including strains that were not susceptible to BCP01 (Table 1). This distinction likely reflects the different biological requirements of whole-phage infection and exogenously applied endolysin activity, because the former depends on multiple sequential infection steps whereas the latter acts directly on the exposed peptidoglycan of Gram-positive bacteria, although access and binding may still be influenced by strain-specific cell wall features such as teichoic acids and surface-associated polymers [17, 36]. A similar pattern has been reported for other *Bacillus* phage endolysins. For example, although the parental phage PBC1 showed extremely narrow host specificity, its endolysin LysPBC1 displayed a broader lytic spectrum within the genus *Bacillus*, and LysPBC4 was likewise reported to have broader lytic activity than its parental phage PBC4 [30, 37]. In addition, other *Bacillus* phage endolysins such as LysB4 and LysBPS13 have been shown to possess either relatively broad lytic activity or favorable biochemical stability, supporting the view that phage-derived endolysins can extend the functional antibacterial range beyond that of the parental phage particle [38, 39]. In this context, the broader intrageneric activity of LysBCP01 and its retained antibacterial activity in sterile cabbage and milk support its relevance as a phage-derived antimicrobial protein. Moreover, supplementary domain-swapping experiments with LysBCP01- and LysPBC1-derived chimeric endolysins yielded narrower lytic spectra than those of the parental proteins, suggesting that the overall lytic spectrum is shaped by the combined contribution of both the EAD and CBD rather than by either domain alone. It should also be noted that the concentrations of LysBCP01 used in the food application assays were relatively high. However, this is not unexpected for protein-based antimicrobials in complex food matrices, where diffusion limitation, adsorption to food components, and reduced access to target cells can substantially diminish apparent activity compared with buffer-based assays [7, 22]. Similar considerations have been noted in previous studies of phage endolysins applied to food-associated systems [7, 22]. Therefore, the present food application data should be interpreted primarily as proof-of-concept evidence supporting the feasibility of LysBCP01 in model food matrices, while further optimization will be necessary for practical application.

In addition to its antibacterial activity, the modular organization of LysBCP01 also enabled functional separation of catalytic and recognition roles. Whereas full-length LysBCP01 acted as a lytic antimicrobial protein, EGFP-LysBCP01_CBD functioned as a host recognition probe with a binding range identical to the lytic spectrum of LysBCP01. This result is consistent with the established view that, in Gram-positive phage endolysins, the C-terminal CBD largely determines cell wall recognition specificity, while the N-terminal catalytic domain mediates peptidoglycan hydrolysis [17, 40]. The detection performance of EGFP-LysBCP01_CBD is also in line with previous studies showing that phage endolysin-derived CBDs can serve as highly specific diagnostic tools for Gram-positive bacteria, including *B. cereus* [41, 42]. In particular, the CBD derived from the *B. cereus* phage PBC1 was previously shown to function as a selective biological probe for *B. cereus*, supporting the interpretation that CBD-mediated recognition can be exploited independently of lytic activity [41, 42]. In this context, the ability of EGFP-LysBCP01_CBD to retain binding activity under a broad range of pH and temperature conditions and to detect *B. cereus* in milk within a short incubation time further supports the applicability of the LysBCP01-derived CBD as a practical recognition element for rapid detection in food-associated environments. Thus, the same phage-derived scaffold could be functionally separated into a lytic antimicrobial format (LysBCP01) and a non-lytic recognition format (EGFP-LysBCP01_CBD).

Although the present study demonstrated the applicability of BCP01, LysBCP01, and EGFP-LysBCP01_CBD under representative food-associated conditions, several limitations should be acknowledged. First, the food application experiments were conducted in model systems using sterile cabbage and milk artificially inoculated with *B. cereus*, and therefore do not fully reflect the microbiological and physicochemical complexity of naturally contaminated foods. Second, the present work focused primarily on vegetative cells, whereas the persistence of *B. cereus* in foods is also closely associated with spore formation and toxin production [2, 43]. In addition, although EGFP-LysBCP01_CBD enabled fluorescence-based detection of *B. cereus* in milk, the assay was evaluated only at a qualitative level. Accordingly, further studies will be needed to determine quantitative performance parameters, including the detection limit, analytical sensitivity and specificity, and applicability in more complex food systems or sensor-integrated formats. Future work should also evaluate the performance of BCP01 and LysBCP01 in a broader range of food matrices and examine their effects on spores and toxin-associated risk.

## Supplemental Materials

Supplementary data for this paper are available on-line only at http://jmb.or.kr.



## Figures and Tables

**Table 1:** Host range of BCP01, LysBCP01 and EGFP-LysBCP01_CBD1.