# Supplementary material for: Bacteriophage BCP01 and Its Endolysin as Tools for Biocontrol and Rapid Detection of Bacillus cereus
Source: J Microbiol Biotechnol. 2026 Apr 24;36:e2604006. doi: 10.4014/jmb.2604.04006 (PMC13125745; doi:10.4014/jmb.2604.04006)
Supplement: Supplementary file 1 [file jmb-36-e2604006-supple.pdf]

J. Microbiol. Biotechnol. 2026. 36: e2604006
https://doi.org/10.4014/jmb.2604.04006

Bacteriophage BCP01 and Its Endolysin as Tools for Biocontrol and Rapid Detection of *Bacillus cereus*

You-Tae Kim[a,b,c,‡], Ji-Eun See[d,‡], and Ju-Hoon Lee[a,b,c,d*]

[a]Department of Agricultural Biotechnology, Seoul National University, Seoul, Republic of Korea
[b]Department of Food and Animal Biotechnology, Seoul National University, Seoul, Republic of Korea
[c]Center for Food and Bioconvergence, Seoul National University, Seoul, Republic of Korea
[d]Department of Food Science and Biotechnology, Kyung Hee University, Youngin, Republic of Korea

[*]**To whom correspondence may be addressed:** Dr. Ju-Hoon Lee.
E-mail: juhlee@snu.ac.kr

[‡]These authors have contributed equally to this work

Supplementary Table and Figures

Table S1. Host range of LysBCP01, LysPBC1, BCP01EAD-PBC1CBD and PBC1EAD-BCP01CBD chimeric proteins[a]

Bacteria	LysBCP01	LysPBC1	BCP01EAD-PBC1CBD	PBC1EAD-BCP01CBD
Bacillus cereus ATCC 10876	+++	++	+++	+++
ATCC 13061	-	+++	-	-
ATCC 14579	+++	++	++	+
ATCC 21768	+++	+++	+++	++
ATCC 21772	+++	+++	+++	+++
ATCC 27348	++	+++	+++	+++
B. circulans JCM2504	-	+	-	-
B. licheniformis JCM 2505	++	+	-	++
B. megaterium JCM 2506	+	+	-	-
B. mycoides ATCC 6452	-	++	-	-
B. pumilus JCM 2508	-	-	-	-
B. sphaericus JCM2502	+++	+	++	-
B. subtilis ATCC23857	-	+	-	-
B. thuringiensis ATCC10792	+++	++	+++	+++
Enterococcus faecium ATCC27276	-	-	-	-
Listeria monocytogenes ATCC 15313	-	-	-	-
Staphylococcus aureus ATCC 29213	-	-	-	-
Yersinia enterocolitica ATCC 55075	-	-	-	-

[a], The relative lytic activity of endolysin was obtained by measuring the percent drop in OD_{600} in 8 min. -, no lysis; +, limited lysis; ++, medium lysis;+++, rapid lysis.

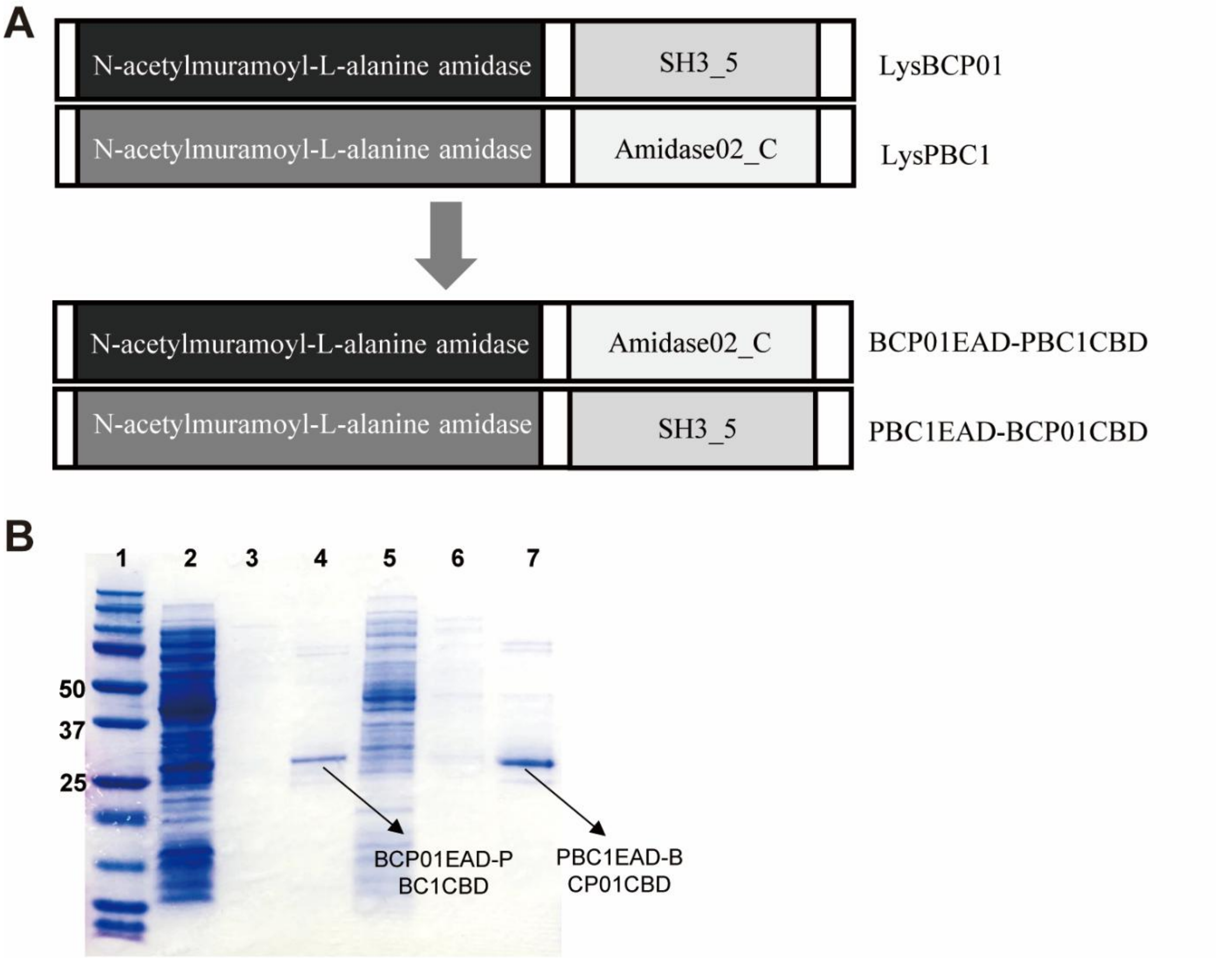

Fig. S1. Construction and purification of chimeric endolysins derived from LysBCP01 and LysPBC1. (A) Schematic diagrams of BCP01EAD-PBC1CBD and PBC1EAD-BCP01CBD. **(B)** SDS-PAGE analysis of purified chimeric proteins.

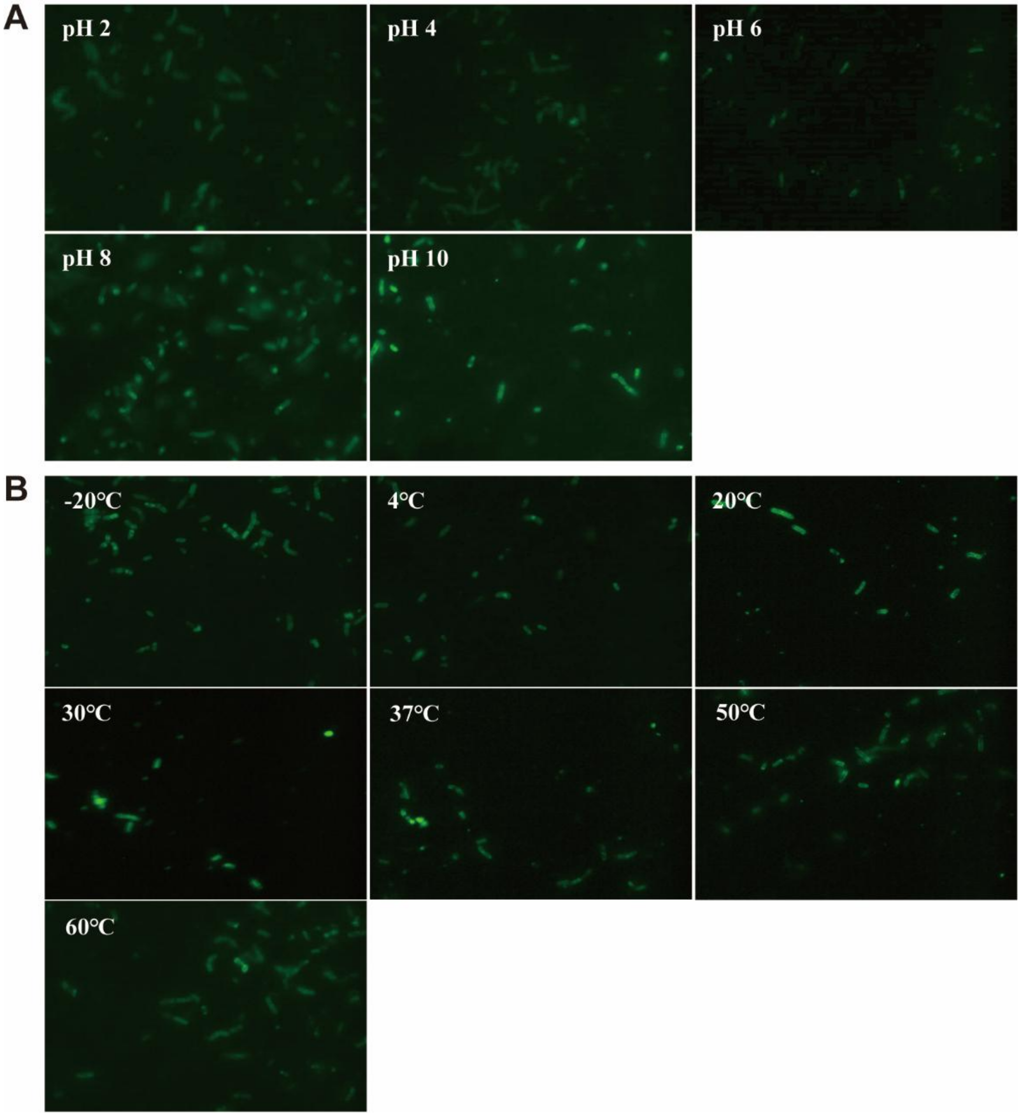

Fig. S2. Stability of EGFP-LysBCP01_CBD binding activity under different pH (A) and temperature (B) conditions against *B. cereus* ATCC 14579.

41
